# First report of the leaf-mining genus *Antispila* Hübner, [1825] from mainland China, with the description of a new species feeding on *Cornus* (Lepidoptera, Heliozelidae)

**DOI:** 10.3897/zookeys.686.13680

**Published:** 2017-07-24

**Authors:** Tengteng Liu, Shuxia Wang

**Affiliations:** 1 College of Life Sciences, Shandong Normal University, Jinan 250014, China; 2 College of Life Sciences, Nankai University, Tianjin 370001, China

**Keywords:** *Antispila*, China, *Cornus walteri*, DNA barcode, Heliozelidae, Lepidoptera, new record, new species

## Abstract

The genus *Antispila* Hübner, [1825] is newly recorded from mainland China. *Antispila sinensis* **sp. n.**, the first formally named Heliozelidae species in mainland China, is described. The host plant of the new species, *Cornus walteri* Wangerin (Cornaceae), is widespread through east Asia and is used as a shade tree in city parks in Jinan City, where the moth causes damage to foliage. Morphological and molecular analyses distinguish *A. sinensis* **sp. n.** from its close relatives. The adult, pupa, larva, host plant, leaf-mines, and the shield of the new species are illustrated, as are male and female genitalia, venation, and larval chaetotaxy. DNA barcodes of the new species are also provided.

## Introduction

Leaf-mining moths, small and large (Liu and Yan 2017), are poorly investigated in China and those in the family Heliozelidae are no exception. Unlike the situation in Europe and North America, Chinese leaf-mining moths are generally known only from their capture at light and a more comprehensive investigation by rearing is required.

The family Heliozelidae comprises 125 described species in 12 genera, with the largest diversity in North America and Australia (van Nieukerken et al. 2011, 2012, 2015). Although an unidentified *Heliozela* sp. from Taiwan is listed in a checklist (Heppner and Kuroko 1992) and seven DNA barcode records of Heliozelidae from Taiwan, including *Antispila* species, are present in the BOLD database (BOLD: www.boldsystems.org), no Heliozelidae species has yet been recorded from mainland China.

The genus *Antispila* Hübner, [1825], comprising some 40 species, is widely distributed worldwide, but most diverse and best studied in the Palaearctic and the Nearctic regions (Kuroko 1961, 1987, Lee et al. 2006a, Lee et al. 2006b, van Nieukerken et al. 2012). Nevertheless, it has not been recorded in mainland China until the present. Vitaceae and Cornaceae comprise the major host plant families (van Nieukerken et al. 2012, Regier et al. 2015).

During collecting trips targeting leaf-mining moths, an abundant population of *A. sinensis* sp. n. was found in Mt. Fohui Park in Jinan City, Shandong Province, where it damaged its host plant *Cornus walteri* Wangerin (Cornaceae). *Cornus walteri* is native and is frequently planted in Mt. Fohui Park, and plays an important role as a shade tree along footpaths. Another population of *A. sinensis* sp. n. was found on Mt. Baxian, Tianjin City, where less damage to its host plant was observed.

In the present paper, the new species *A. sinensis* sp. n. is described and the moth’s morphology, life-history, hostplant, and DNA barcode is reported upon. The potential for this moth to damage its host plant *Cornus walteri* is described.

## Material and methods

Leaves with active mines were placed in small plastic bags for rearing. After the shields had been exscinded and the larvae had left the mines, the larval shields were transferred into closed containers and leaves with vacant leaf-mines were dried in a plant press. The larval shields, the corresponding adults, and the vacant leaf-mines were identically coded.

Genitalia and wings were dissected and mounted according to the methods introduced by Li (2002), but stained with Eosin Y and/or Chlorazol Black. Illustrations were prepared by using a Leica DM750 microscope. Line drawings were refined in Photoshop® CS4 software. Adult photographs were taken with a Leica M205A stereo microscope and a Leica S6D stereo microscope. Photographs of host plant and leaf-mines were taken in the field using a Canon EOS camera. The larva was macerated in 10% KOH, boiling for 10 min, rinsed in water and alcohol, then stained with Chlorazol Black, and kept in glycerin prior to examination. Measurements of shields were carried out using an ocular micrometer on an Olympus SZ11 stereo microscope.

DNA was extracted from adult specimens preserved in 95% ethanol in Shandong Normal University, Jinan, China (SDNU), with genitalia and wings preserved as vouchers (Knölke et al. 2005). Total DNA was extracted using the TIANamp Micro DNA Kit (Code: DP316, Tiangen, China). Fragments of the mitochondrial COI gene were amplified using the primer pair LEPF1/R1 and the protocol introduced by Hebert et al. (2003). The sequence data used in this study have been deposited in the public Dataset “DS-ANTISIN” in the BOLD database. Sequences were aligned using MUSCLE model and genetic distance estimation was analyzed using Kimura 2-Parameter model in the BOLD.

Terminology of adult follows van Nieukerken et al. (2012), and that of larva follows Lee et al. (2006) and Low (2008) with some modifications. Thoracic segments I–III and abdominal segments 1–10 are abbreviated as TI–TIII and A1–A10, respectively. The classification of the host plant is based on APG (2016).

The holotype of the new species is deposited in the Insect Collection of Nankai University (NKU), and paratypes are distributed in NKU and SDNU as stated in the text.

## Taxonomy

### Antispila sinensis sp. n.

Taxon classificationAnimaliaLepidopteraHeliozelidae

http://zoobank.org/2529442C-31AC-41AE-AB57-5093C72EFFC1

Figs 1–3 , 4–6 , 7 , 8–12 , 13–14 , 15–22 , 23

#### Diagnosis.

This species can be easily separated from the two European *Cornus* feeding species, *A. treitschkiella* (Fischer von Röslerstamm, 1843) and *A. metallella* (Denis & Schiffermüller, 1775), by the forewing with the opposite basal spots separated and thus not forming a fascia, and by the vein Rs_3+4_ reaching costa near apex and the absence of the stem of vein M. *Antispila treitschkiella* has a basal fascia in the forewing, and *A. metallella* has the vein Rs_3+4_ reaching termen and has an M stem in the forewing. Specimens with the inner spots joined into a fascia, *A. sinensis* could be separated from *A. treitschkiella* reliably by genitalia. Although DNA Barcode distance analysis suggests *A. tateshinensis* Kuroko, 1987 and *A. purplella* Kuroko, 1961 to be the closest relatives (Fig. 23), morphologically, *A. sinensis* most closely resembles *A. purplella*, which feeds on *Cornus controversa* and *C. brachypoda*. These two species are similar in terms of the hooked structure of the phallus, the cusps of the ovipositor and wing venation but can be distinguished by the thin ventral tube in the distal part of the phallus, which is bifurcated in *A. purplella*. In addition, the region of the vinculum adjacent to the triangular median protrusion is almost flat in *A. sinensis*, while it is apparently concave in *A. purplella*.

#### Type material.

China: **Holotype** ♂, Mt. Baxian, 40.18°N, 117.55°E, 300–600 m, Ji County, Tianjin, larva coll. 5.ix.2013, emerged 9.iii.2014 (indoors), leaf-mine on *Cornus walteri*, leg. Tengteng Liu, genitalia slide No. LIU12870 (deposited in NKU).

**Paratypes. Tianjin**: 2♂, 1♀, larva coll. 5.ix.2013, emerged 8, 9.iii.2014, 1♂, 1♀, larva coll. 6.ix.2013, emerged 6, 12.iii.2014, 3♂, 1♀, larva coll. 9.viii.2013, 20, 25.x.2013, 10, 11.iii.2014, 5♂, 5♀, larva coll. 24.vi.2014, emerged vii-2014, other data same as holotype, genitalia slide Nos. LTT12559♀, LTT12613–4♂, LTT12866♀, LTT12867–9♂, LTT12871–3♂, LTT12874–5♀, wing slide Nos. LTT12613–4W, LTT12874W (deposited in NKU); 12♂, 12♀, Mt. Baxian, 40.194°N, 117.557°E, 300 m, Ji County, Tianjin, larva coll. 29, 30.vi, 23.vii.2015, shield made 2, 3, 5, 25, 27.vii.2015, emerged 27, 29, 31.vii, 2, 13, 20.viii.2015, leaf-mine on *Cornus walteri*, leg. Tengteng Liu, registered Nos. SDNU_BXS150601–05, BXS150607, BXS150608–12, BXS150615, BXS150617–19, BXS150625–27, BXS150630–31, BXS150633, BXS150701–03, BXS150704–05 (deposited in SDNU); **Shandong**: 7♂, 6♀, Mt. Fohui, Jinan, 36.633°N, 117.050°E, 260 m, leaf-mine on *Cornus walteri*, larva coll. 7.ix.2015, shield made 7, 10, 11, 13.ix.2015, emerged 26, 30.x.2015, 31.iii, 15, 19, 23, 24.vi.2016, leg. Tengteng Liu, genitalia slide No. LIU15003♂, registered Nos. SDNU_JN150903–04, JN150909, JN150911–14, JN150918–19, JN150926, JN150928–9, JN150939, JN150944, JN150947 (deposited in SDNU).

**Figures 1–3.**
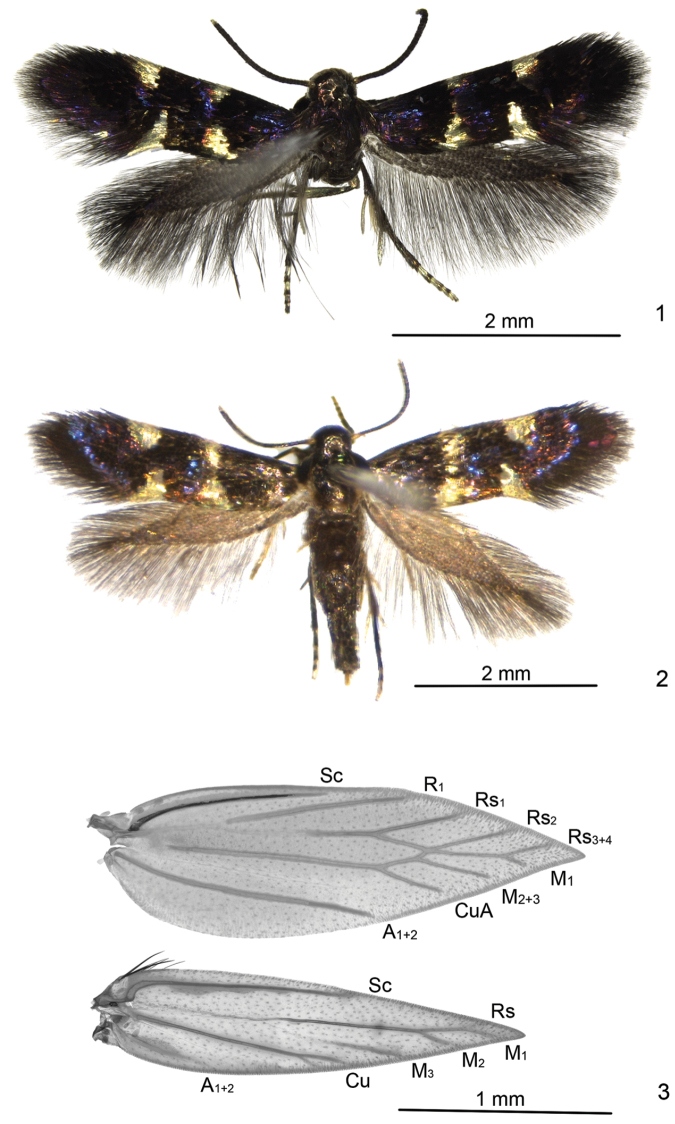
Adult and wing venation of *Antispila sinensis* sp. n. **1** Adult, paratype, male, BXS130941 **2** Adult, paratype, female, SDNU_JN150947 **3** Wing venation, female, slide No. LTT12874W, paratype.

**Adult** (Figs 1, 2). Forewing length 2.4–3.0 mm. Head deep silvery gray, smooth. Base of proboscis covered with larger scales. Labial palpus dark gray. Antennae blackish fuscous, distal two segments grayish white. Thorax and tegula dark gray, with purplish-blue metallic reflection. Legs silvery gray to deep silvery gray, tarsi dark gray dorso-distally except last segment. Forewing black, with purplish-blue reflection; two more or less triangular silvery white spots on costa at 1/3 and 2/3; two similar spots on dorsum opposite costal spots, inner pair sometimes joined into a fascia, especially in female (Fig. 2); cilia black basally, dark gray distally. Hindwing dark gray. Abdomen dark gray dorsally, silvery gray ventrally; vestiture on external genitalia dark gray.

*Venation* (Fig. 3). Forewing with Sc reaching before middle on costa; R_1_ from 2/5 on upper margin of cell to costal 3/5, Rs_1_ from 1/7 on upper margin of cell to costal 3/4, Rs_2_ from distal end of cell, Rs_3+4_ reaching costa before apex; cell triangular distally; M_1_ stalked with Rs_3+4_, to termen near apex, M_2+3_ from near distal end of cell; Cu from distal 1/7 of lower margin of cell; A_1+2_ to beyond middle of dorsum. Hindwing with Sc to costal 3/5, R+M ending in 4 branches: Rs to costa near apex, M_1_, M_2_ and M_3_ to dorsum; Cu ending in 2 branches; A_1+2_ weak. Male with one frenulum, female bearing two frenular bristles and several hairscales.

**Figures 4–6.**
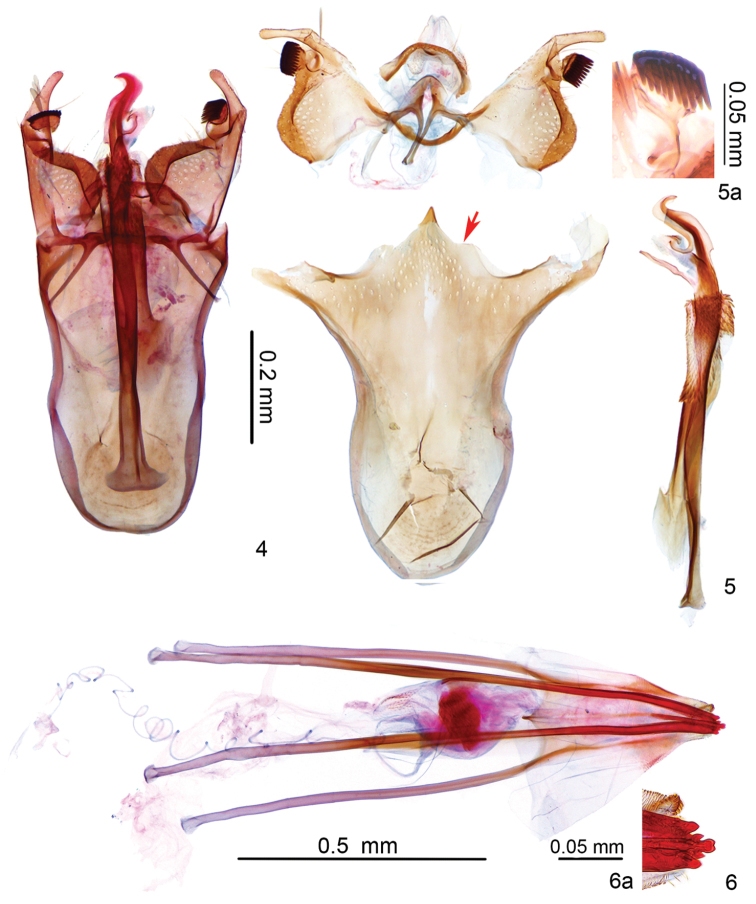
Genitalia of *Antispila sinensis* sp. n. **4** Male genitalia *in situ*, slide No. LTT12873, paratype **5** Male genitalia unrolled, slide No. LTT12872, paratype, red arrow referring to the region of the vinculum adjacent to the triangular median protrusion **5a** Close-up of comb teeth on pecten, slide No. LIU15003 **6** Female genitalia, slide No. LTT12875, paratype **6a** Close-up of ovipositor tip, same slide as in Fig. 6.

*Male genitalia* (Figs 4, 5, 5a). Uncus setose on posterior margin, with two setose sacs ventrally. Vinculum slightly shorter than phallus, anteriorly rounded, posteriorly almost even besides triangular median protrusion. Valva more or less rectangular on basal half, digitally extended distally, pecten on pedicel, with 10–14 comb teeth (Fig. 5a); transtilla almost uniform in width, sublateral processes relatively short. Juxta about half as long as phallus, densely covered with teeth on basal 2/5, anteriorly spade-shaped. Phallus long, strongly expanded at base laterally; distal part beyond base of juxta complex, curved, with small hooks distally and medio-ventrally, an incision before middle dorsally, a thin tube extruded near base ventrally.

*Female genitalia* (Fig. 6, 6a). Ovipositor with 3 cusps at either side (Fig. 6a). Eighth sternum medially indented posteriorly, with many papillate setal sockets, triangularly protruded on posterior margin ventrally. Vestibulum oval, covered with spines anteriorly, inner with paired wrinkled sclerotization, each with a long spine. Corpus bursae membranous.

**Figures 7.**
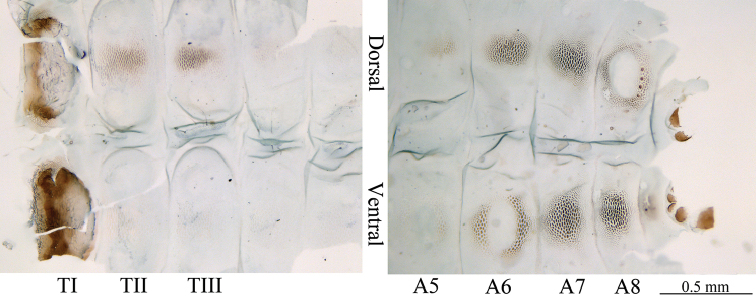
Final instar larva in leaf-mine, showing sclerotized plate and granules on the body, registered No. SDNU_JN150907.

**Figures 8–12.**
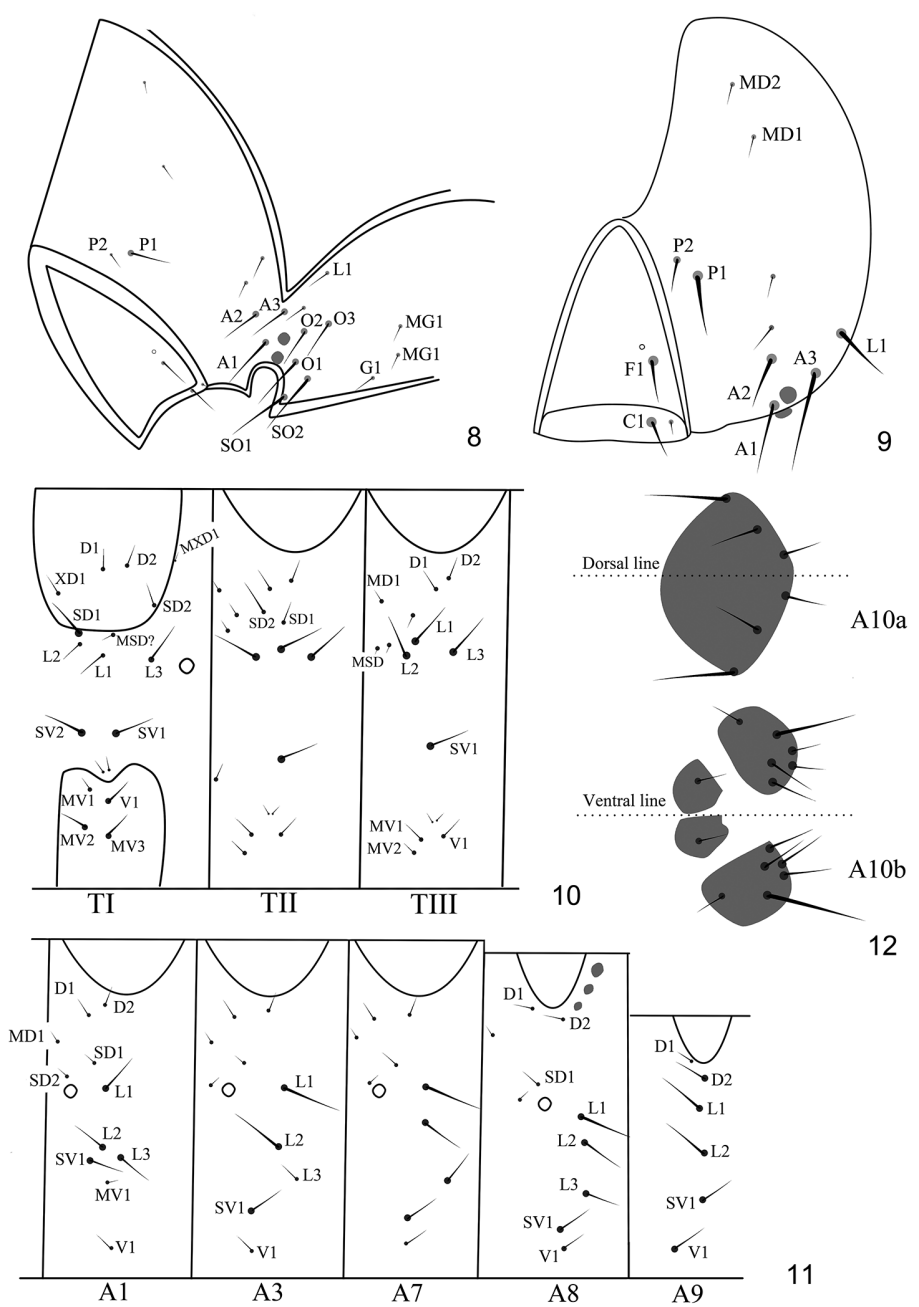
Larval chaetotary of *Antispila sinensis* sp. n. **8** Head lateral view **9** Head front view **10** Thorax **11** Abdomen **12** A10 dorsal and ventral view.

**Final instar larva in mine** (Figs 7–12). Body length about 5.0 mm. Two stemmata visible. Prothorax (TI) with sternum and tergum strongly sclerotized (Fig. 7). T2–3 and A5–9 with terga strongly sclerotized, covered with granules; eighth tergum with a weakly sclerotized oval zone medially, a row of sclerotized pointed bumps along posterior margin (Fig. 7). A5–9 with sternum strongly sclerotized, covered with granules, A5 and A6 with weak sclerotized zones medially (Fig. 7). Tenth tergum more or less oval, lateral and ventral plate sclerotized. Thorax legs and prolegs absent (Fig. 7).

*Head* (Figs 8–9). Three long setae on A-group, A1 internal to stemmata, A3 posterior to stemmata I, A2 internal to A3; Fa internal and dorsal to F1; P-group close to adfrontal area, with P1 longer than P2, two short setae lateral and ventral to P1 which might be real P setae, while P setae here designated might be AF setae; L1 posterior to A3; V-group with 2 micro-setae; O1 ventral to stemmata II, O2 and O3 ventral to stemmata I; SO1 below antennae, SO2 dorsal to SO1; G-group with three setae, G1, MG1, and MG2.

*Thorax* (Fig. 10). TI with D1 anterior and slightly ventral to D2, XD1 anterior and ventral to D1, near anterior margin prothoracic shield, MXD1close to posterior margin of prothoracic shield; SD1 and SD2 on lateral margin and posterior margin respectively, SD1 longer than SD2; L-group with 3 setae, L2 ventral to SD1, L3 close to spiracle, L2 between L1 and L3; SV-group with two setae, same in length; two micro-setae on concave of lateral margin of sternum; sternum with 4 MV setae. TII similar to TI, one MD setae anterior to D1; SD2 close to SD1, with two MSD setae anteriorly; SV-group with only one seta; two MV setae exist. TIII similar to TII, except with SD2 missed.

*Abdomen* (Figs 11, 12). A1 with D-group and SD-group setae very short, SD1 dorsal and posterior to spiracle, SD2 next to spiracle; L-group and SV-group setae long, L1 posterior to spiracle, L2 ventral to L1, L3 posterior and ventral to L2, SV1 anterior and slightly ventral to L3. A2 similar to A1. A3 similar to A1, but with L3 shorter, SV1 moved ventrally, MV1 absent. A4–7 similar to A3, except with L3 longer on A7. A8 with D1 anterior and dorsal to D2, L-group more or less same in length, arranged in line. A9 similar to A8, but with L3 absent. A10 apparently sclerotized on both sternum and tergum, with a single plate dorsally and two paired plates ventrally (Fig. 12).

**Figures 13–14.**
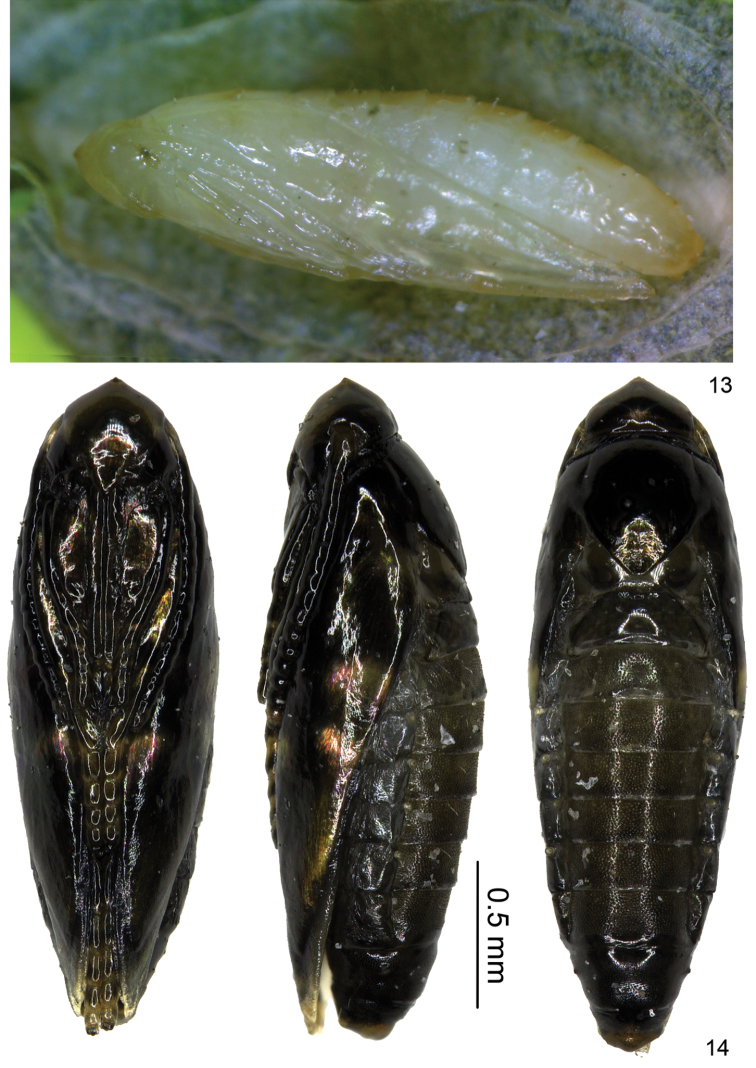
Pupa of *Antispila sinensis* sp. n. **13** a white early stage pupa **14** pupa just before emergence in ventral, lateral, and dorsal view.

**Pupa and shield** (Figs 13–14, 20–22). Shield 4.3 ± 0.3 mm in longitudinal diameter, 2.4 ± 0.2 mm in latitudinal diameter, with 13.2 ± 3.3 spines (n = 23), but also some extremes without spines (Fig. 21). Pupa changing from white to dark fuscous during maturity (Figs 13–14).

**Figures 15–22.**
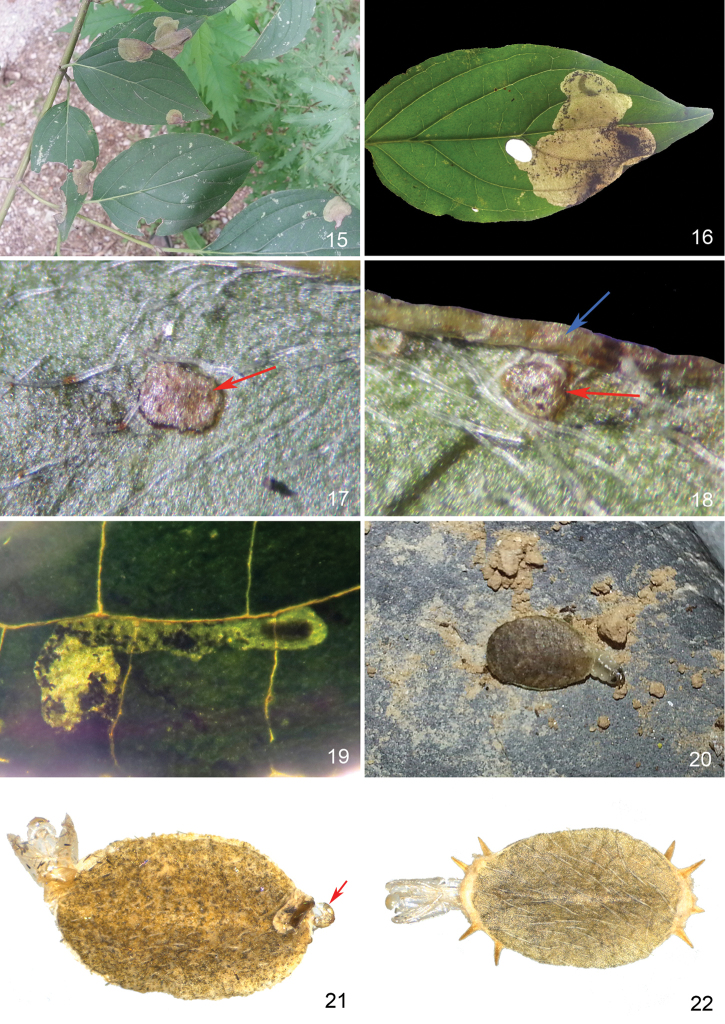
Leaf mine, host plant and shield of *Antispila sinensis* sp. n. **15** Leaf mines on *Cornus walteri* **16** A large blotch mine occupied by two larvae one of which has made a cut-out **17** piercing scar on the disc of a leaf indicated by a red arrow **18** piercing scar (red arrow) near margin of a leaf where callus also occurs (blue arrow) **19** Early linear mine with an active larva **20** Larva in freshly made shield, searching for a pupation site on the ground **21** Shield without spines along margin, pupal exuviae and exuviae of prepupa (red arrow) **22** Shield with several spines along margin, pupal exuviae.

#### DNA barcode.

**Figure 23.**
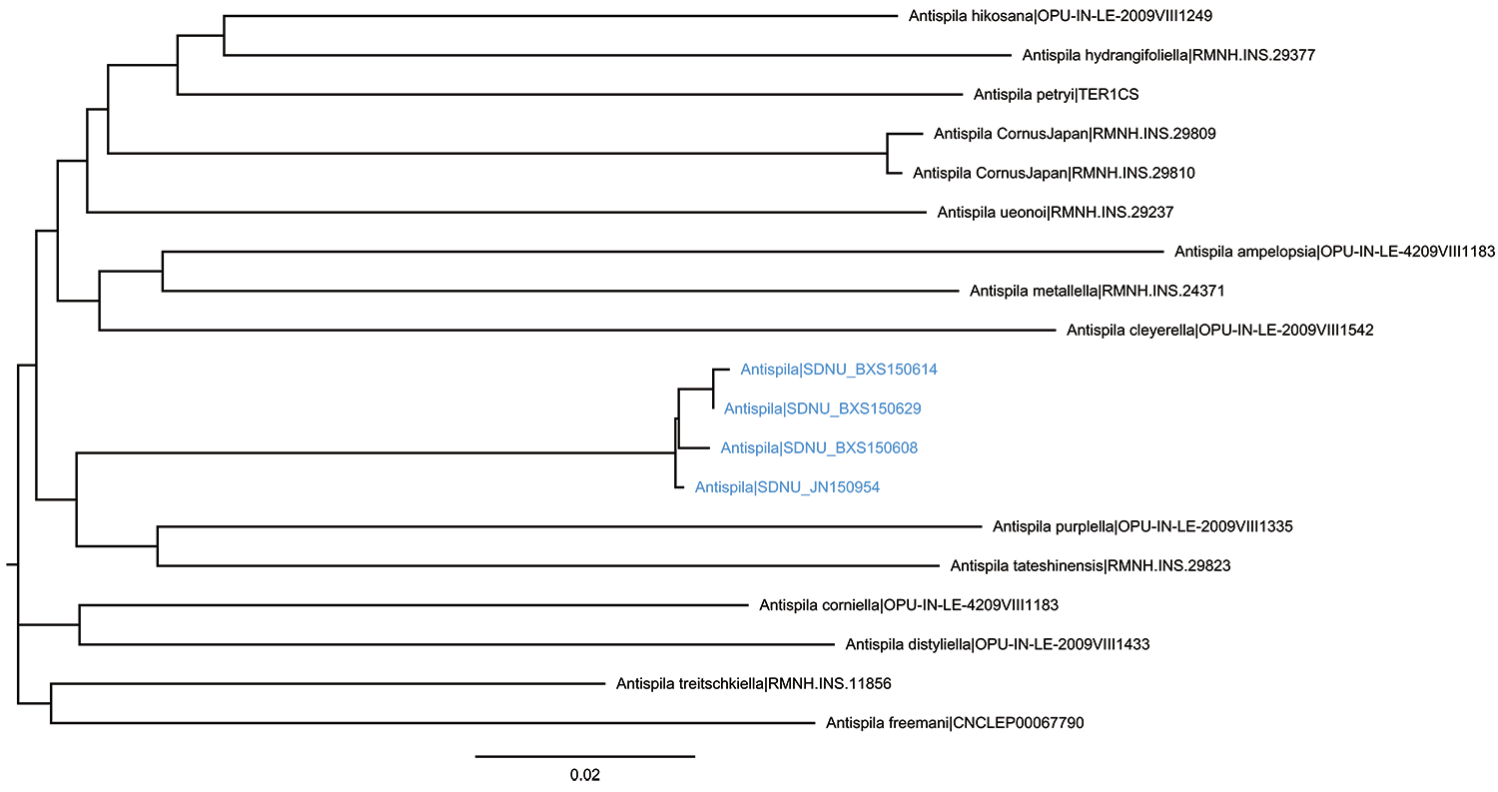
Neighbor-joining tree estimated from DNA barcode sequences under dataset “DS-ANTISIN”, performed by using Kimura 2-Parameter model. Clades representing *A. sinensis* sp. n. are marked blue.

Four DNA barcodes from individuals from each of the collecting sites have been submitted to BOLD under the public Dataset “DS-ANTISIN” (doi: 10.5883/DS-ANTISIN). The Barcode Identification Number is BOLD:ADG5043. A neighbor joining tree with barcodes of other *Antispila* species, particularly *Cornus* feeding species and Japanese species, is given in Fig. 23.

#### Host plant.

*Cornus walteri* Wangerin (Cornaceae).

#### Distribution.

China (Shandong, Tianjin).

#### Biology.

Egg is laid on the lower side of a leaf, usually close to leaf margin and occasionally near main vein, where a darker area of callus (piercing scar) caused by piercing of the ovipositor is invariably seen (Figs 17, 18). The leaf-mine begins as a line and extends along veins if it cannot go through (Fig. 19). The mine soon enlarges to a blotch, incorporates most or all of the earlier linear mine and finally, ends in a cut-out (Fig. 16). Several mines can be found on the same leaf (Figs 15, 16). Frass primarily occupies the opposite side of the cut-out in the mine, with a small amount extending adjacent to the cut-out (Fig. 16). The newly made larval shield often falls onto the ground, after which the larva searches for a place to pupate (Fig. 20). The larva molts once in the shield before pupation and the final larval exuviae are often protruding at one end of the shield, opposite to the end where the pupal exuviae protrude (Fig. 21). This species overwinters as a prepupa in the shield and pupates during early spring to emerge later as an adult. At least three generations occur annually.

#### Etymology.

The specific name is derived from the country where the new species represents the first formally named Heliozelidae species.

## Supplementary Material

XML Treatment for Antispila sinensis
